# Operation for Varicocele by the Subcutaneous Wire-Loop

**Published:** 1875-09

**Authors:** 


### OPERATION FOR VARICOCELE BY THE SUBCUTANEOUS WIRE-LOOP.

Mr. Rich. Barwell, Surgeon to Charing Cross Hospital, advocates the treatment of varicocele by the method which he describes as follows: First, it is most important that the vas deferens and spermatic artery be separated from the veins. This is best done by the surgeon standing on the patient's left (supposing the disease to be on that side), and taking that half of the scrotum, about an inch above the testicle, between the left finger and thumb, and feeling for the duct by rubbing the two surfaces of the bag gently together while its walls glide from his grip. When he has thus brought the duct and artery from among the venus plexus, and holds them between his thumb and finger-tip, he must let them slip away to the back, so that he will enclose the veins within the circle of his finger and thumb, and exclude from it the artery and the vas deferens. The digit tips and slightly compressed scrotum will thus separate the one set of vessels from the other. If, in seeking for the duct, the surgeon has allowed a few veins to slip away with it, he can begin again, with the advantage of having no longer the whole mass, but only a few vessels, to deal with. If he be not sure of having successfully isolated this important part, he must examine the condition of things with his right hand while keeping the left in the same position. Supposing this separation accomplished, the bit of scrotum between the finger-tips is to be squeezed rather tight, and a needle, armed with iron or silver wire, not too fine, thrust straight through above them. Now the part may be released from the grasp, and the needle passed in again at the puncture of exit, in front of the veins, and out at the first place of entrance. Thus at one opening protrude the two ends of the wire, and at the other a loop; by drawing on the ends, the loop is pulled into the scrotal cavity, and closely surrounds the varicocele. Each end is passed through a hole in an instrument, which Mr. Barwell has described in a former paper, and drawn tight enough to make the veins below swell, bulge, and partly consolidate. Every other day, or every day, if time be an object, the wire may be tightened until it has cut through all the consolidated veins, and has come away. No suppuration nor apparent inflammation accompanies the process. The patient need not be confined to his bed after the first forty-eight hours.

Mr. Barwell says that one caution must be given. In passing the needle the second time, *i e.*, in front of the veins, its course must be entirely in the cavity of the scrotum. If, from over anxiety to include every vein, or, from other cause, some of the lining fascia be strangulated in the loop, the case will be considerably retarded, and some amount of suppuration, accompanied by swelling in the walls of the sac, is likely to ensue. He says that when the operation is well performed, there is but little pain accompanying or following it, and in some cases where pain in the testicle had previously been severe, relief was the immediate result.

Mr. William Rose, in a note to *The Lancet* in regard to Mr. Barwell's article, says that Mr. Wood, of King's College Hospital, for the past nine years has operated for varicocele by a method which Mr. Rose thinks superior to that employed by Mr. Barwell, in that the ends of the wire loop that surround the spermatic veins are passed through an instrument devised by Mr. Wood, so that the loop is steadily tightened by means of the constant action of a spring. In this way Mr. Rose says that the susequent daily tightening of the wires, according to Mr. Barwell's plan, is avoided, and he further says that the veins are cut through in a much shorter time. He refers to *The Lancet* of July 4, 1868, for an account of the operation, and a wood-cut of the instrument.—*The Lancet*, June 12 and 19, 1875.—*Medical Record.*

